# Far from home: the role of glial mRNA localization in synaptic plasticity

**DOI:** 10.1261/rna.079422.122

**Published:** 2023-02

**Authors:** Dalia S. Gala, Joshua S. Titlow, Rita O. Teodoro, Ilan Davis

**Affiliations:** 1 Department of Biochemistry, The University of Oxford, Oxford OX1 3QU, United Kingdom; 2 iNOVA4Health, NOVA Medical School—Faculdade de Ciências Médicas, Universidade Nova de Lisboa, Lisboa 1169-056, Portugal

**Keywords:** glia, mRNA localization, localized translation, neuronal synaptic plasticity

## Abstract

Neurons and glia are highly polarized cells, whose distal cytoplasmic functional subdomains require specific proteins. Neurons have axonal and dendritic cytoplasmic extensions containing synapses whose plasticity is regulated efficiently by mRNA transport and localized translation. The principles behind these mechanisms are equally attractive for explaining rapid local regulation of distal glial cytoplasmic projections, independent of their cell nucleus. However, in contrast to neurons, mRNA localization has received little experimental attention in glia. Nevertheless, there are many functionally diverse glial subtypes containing extensive networks of long cytoplasmic projections with likely localized regulation that influence neurons and their synapses. Moreover, glia have many other neuron-like properties, including electrical activity, secretion of gliotransmitters and calcium signaling, influencing, for example, synaptic transmission, plasticity and axon pruning. Here, we review previous studies concerning glial transcripts with important roles in influencing synaptic plasticity, focusing on a few cases involving localized translation. We discuss a variety of important questions about mRNA transport and localized translation in glia that remain to be addressed, using cutting-edge tools already available for neurons.

## INTRODUCTION

Across different living systems, mRNA localization is an important mechanism for achieving functional regionalization and polarization of diverse cell types (Das et al. 2021). Domain specific mRNA localization enables local production of several proteins required for specialized structural adaptations and asymmetrical morphology in polarized cells to create regions with distinct functions. Neurons and glia exhibit some of the most extreme forms of polarization, involving the formation of extensive and elaborate cytoplasmic processes. These elongated protrusions extend considerable distances away from the cell body to define numerous independently regulated and unique subcellular environments with specific protein distributions and functions. In neurons, there are many studies demonstrating that such regional specializations are established and maintained either through protein sorting, or through mRNA transport and localized translation (Caceres et al. 1984a,b; Steward and Schuman 2003; Holt et al. 2019; Donlin-Asp et al. 2021; Perez et al. 2021).

One of the most important cellular functions of localized mRNA is to enable synaptic plasticity, a dynamic process that tunes the structure and function of neural circuits to their activity level to enable such diverse processes as brain development and memory formation. Studies of synaptic plasticity have identified thousands of mRNAs that localize to distal compartments of neurons, including *Map2* (Garner et al. 1988), *Camk2a* (Burgin et al. 1990), *beta-actin* (Lawrence and Singer 1986; Bassell et al. 1998) and other cytoskeletal proteins, as well as ribosomes that would be necessary to translate them (Bodian 1965; Steward and Levy 1982; Nedozralova et al. 2022). Neuronal projections are sufficiently long that localized translation can explain how rapid regional production of protein can eliminate the long delay of communicating back to the nucleus to activate gene expression and transport newly synthesized proteins to the periphery and individual synapses. While mRNA localization is widely accepted as a mechanism for synaptic plasticity in neurons, glia, whose role in synaptic plasticity is still emerging, are also likely to require mRNA localization and protein synthesis in synaptic compartments.

What is the role of glia in synaptic plasticity? Traditionally, synapses were described as composed of two main compartments: the pre-synapse specialized in neurotransmitter release, and the post-synapse, responsible for receiving and responding to neurotransmitters, as well as for sending retrograde signals back to the pre-synapse. About 20 years ago, these synapses were redefined as “tripartite,” to include the synaptically associated glial cells (Araque et al. 1999), mainly astrocytes and perisynaptic Schwann cells (PSCs). These, and in fact most glial subtypes are just as polarized as neurons, containing long and numerous cytoplasmic projections that also require individual, region-specific regulation far away from the nucleus. Astrocytes and PSCs had been observed by electron and light microscopy to be in close proximity to synapses, which allows them to influence short-term and long-term plasticity through a diverse array of synaptic functions, including actively influencing synaptic transmission, secreting gliotransmitters and forming phagocytic protrusions needed to eliminate competing axons (Araque et al. 1999; Newman 2003, 2013; Halassa et al. 2007, 2009; Perea et al. 2009; Pérez-Alvarez and Araque 2013; Panatier and Robitaille 2016). Astrocytes have been shown to potentiate medium spiny neurons in a cell-specific manner (Martin et al. 2015), regulate synaptic efficacy in the hippocampus (Panatier et al. 2011), and also regulate dopaminergic transmission in the nucleus accumbens (Corkrum et al. 2020). They are also known to be involved in long-term memory, such as regulation of spatial and contextual memory through potentiation of Schaffer collaterals in the hippocampus (Adamsky et al. 2018), and regulation of fear conditioning through modulation of firing rate in central amydala neurons (Martin-Fernandez et al. 2017). These dynamic processes are likely to require rapid changes in local protein composition, where mRNA localization and localized translation are potential regionalized mechanisms that could provide distal regulation in glial cytoplasmic extensions. However, this topic is far less explored in glia than in neurons and is the central focus of our mini-review.

Here, we first survey the different glial cell subtypes to provide the biological context and then summarize the latest knowledge of distally localized glial transcriptomes and the roles of glial mRNA localization in synaptic plasticity. Then we discuss the key outstanding questions in the field relating to glial mRNA localization and the range of experimental approaches that could be brought to bear on these questions, as has already been achieved in equivalent neuronal studies.

## GLIAL CELL TYPES AND SYNAPTIC PLASTICITY

**FIGURE 1.**
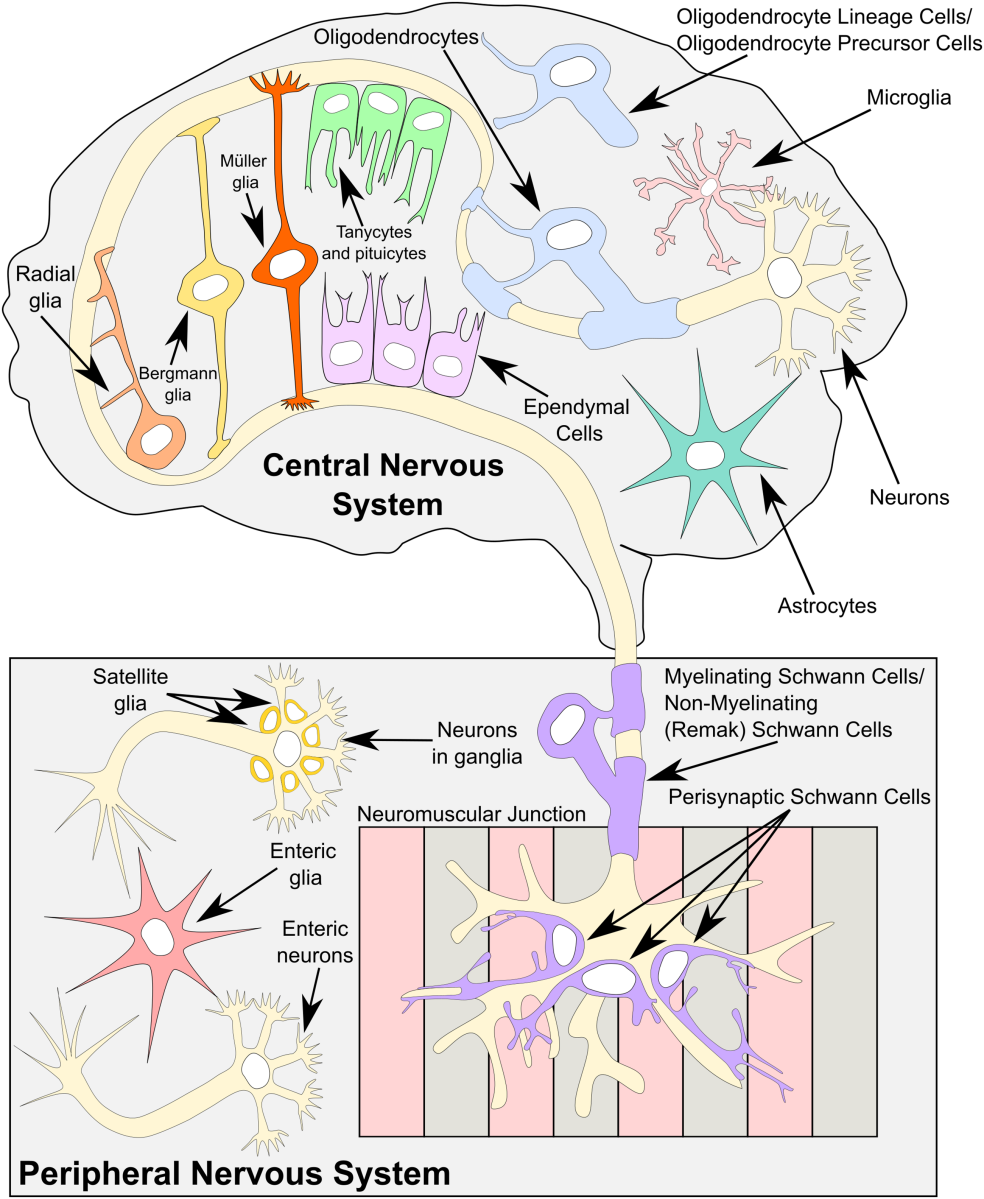
A summary of all major known glial cell types present in both the central and the peripheral nervous systems. The major glia subtypes of the central nervous system are astrocytes and their specialized subtypes are as follows: Bergmann glia in the cerebellum and Müller glia in the retina, oligodendrocytes and their precursors, microglia, ependymal cells, tanycytes, and pituicytes. Radial glia are present in the central nervous system during development, and they differentiate both into neurons and some glial subtypes. In the peripheral nervous system, there are several subtypes of Schwann cells, including myelinating and nonmyelinating Schwann cells, also called Remak Schwann cells and perisynaptic/terminal Schwann cells. Satellite glia surround the cell bodies of the neurons in ganglia, and enteric glia, which are sometimes classed as a Schwann cell subtype, can be found in the enteric nervous system.

Like neurons, glia display a diverse range of morphologies ranging from “immune-cell-like” microglia to cell types with elongated cytoplasmic projections including myelinating oligodendrocytes, Schwann cells, and astrocytes. Such elongated projections can, in the case of astrocytes, contact up to 100,000 neuronal synapses, which can modulate or synchronize the activity of networks of neurons (Ransom and Ransom 2012; Jessen et al. 2015a; Allen and Lyons 2018). Figure 1 provides a graphical representation of the location of all major types of glia throughout the nervous system.

For some time, it has been appreciated that there are a great number of glial subtypes with important roles for synaptic plasticity (Reichenbach et al. 2010). Synaptic plasticity is broadly defined as changes in the strength or efficacy of synaptic transmission at already existing synapses, paired with morphological changes including creation of new synapses, or changes in the size and shape of existing synapses, caused by varying levels of activity. Synaptic plasticity has been proposed to play a central role in the formation of memory and the capacity of the brain to learn and retain information (Citri and Malenka 2008; Harris 2020a,b). Synaptic plasticity requires a series of biochemical and bioelectrical events that lead to changes in the molecular composition of synapses. mRNA localization has long been proposed to be central to this process, as discussed in the introduction. By supplying a pool of mRNAs, ready to be translated when needed, a repertoire of molecules present at the periphery can be modified quickly and efficiently, in response to elevated or reduced activity.

Many studies focused on the role that glial cells could play in the adjustment of the molecular repertoire of the synapses. Astrocytes, for example, have even been dubbed the “master conductors” of the brain because of their control of the formation and function of GABAergic synapses (Takano et al. 2020). Moreover, an increase in astrocytic cAMP, a critical second messenger in neuronal plasticity, was directly demonstrated to modulate memory and induce synaptic plasticity (Zhou et al. 2021). Myelination and oligodendrocyte signaling have long been suspected to play roles in synaptic plasticity (Fields 2005, 2008). The role of glia in regulating plasticity has been recently reviewed (De Luca et al. 2020; Sancho et al. 2021), but the likely involvement of localized translation in regionalized regulation, remains poorly investigated (Blanco-Urrejola et al. 2021; Meservey et al. 2021). Describing the detailed roles of all types of glia is beyond the scope of this introduction, but we provide a summary in Table 1.

**TABLE 1.**
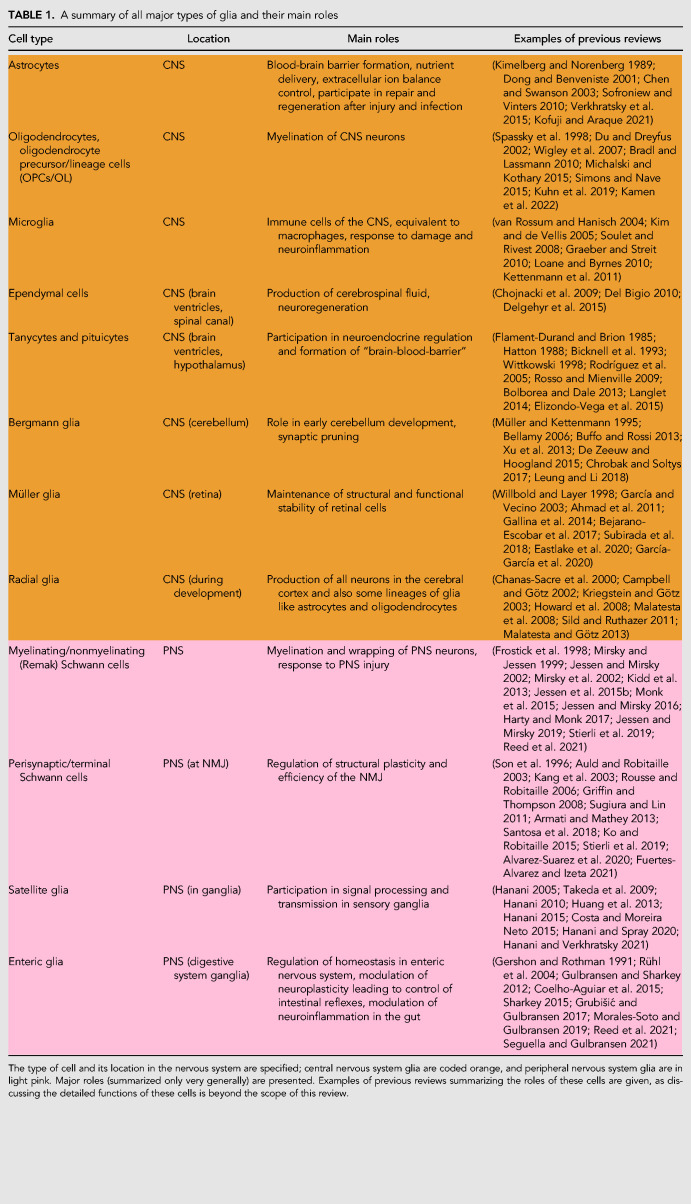
A summary of all major types of glia and their main roles

Localized translation in glial cytoplasmic projections has the potential to modify tripartite synapses very rapidly. The glia present in the tripartite synapses, most notably astrocytes and PSCs, directly influence synaptic functions through their cytoplasmic projections that are in close contact with both pre- and post-synapses. Astrocyte endings near synapses can produce phagocytic protrusions and physically interfere with the synaptic terminals, which would inevitably require cytoskeletal remodeling; astrocytes can also release gliotransmitters and play metabolic and homeostatic roles important for plasticity, all of which have been reviewed (Barker and Ullian 2010; Min et al. 2012; Ota et al. 2013; De Pittà et al. 2016; Zhou et al. 2020; Perez-Catalan et al. 2021; Wahis et al. 2021). Similar observations apply to PSCs. PSCs are capable of extending cytoplasmic finger-like processes into the synaptic cleft, causing a blockage that weakens the nerve-muscle interaction (Smith et al. 2013; Alvarez-Suarez et al. 2020). However, to our knowledge, no studies have been conducted to observe whether any of the described functions are directly dependent on mRNA localization and local translation. In the next sections, we consolidate what is known about the mRNA localization in various types of glia and correlate that with the known roles of glia in regulating synaptic plasticity.

## LOCALIZED GLIAL TRANSCRIPTOMES AND TRANSLATOMES

Single-cell RNA sequencing (scRNA-seq) technology has been widely utilized to define the different glial subtypes and highlight the heterogeneity between them. These studies have recently been reviewed (Sun et al. 2021). scRNA-seq studies provide great insights into the molecular signatures of each glial cell type and the heterogeneity in their transcriptional programs. However, a limitation of those studies, especially in the context of specific regulation of local protein repertoire, is that they do not provide information on transcripts present in glial processes, which are lost during tissue disaggregation required to separate the individual cell bodies (Chen 2005). Similarly, sequencing-based spatial transcriptomics methods lack the resolution required to differentiate individual glial processes. Although imaging-based methods have adequate resolution, particularly in situ sequencing combined with expansion microscopy (Alon et al. 2021), these methods have not yet been extensively applied to glia. Given that most of the glial cytoplasm is present in projections, high resolution imaging-based methods like RNA FISH and the use of fractionation methods are required to define the mRNA repertoire that is present in fine projections of glial cells (Thomsen and Lade Nielsen 2011; Mazaré et al. 2020). Several cell fractionation studies have been published that either identify individual transcripts present at the glial periphery or characterize the entirety of the peripheral glial transcriptomes. Most studies have focused on astrocytes and oligodendrocytes, making them a natural starting point before describing research in other less studied glial cell subtypes.

### The roles of astrocytes in synaptic plasticity

Astrocytes are a glial subtype with an extensive network of cytoplasmic extensions that radiate in all directions from the cell body, leading to their characteristic star-like morphology. They perform crucial roles in synaptic plasticity and blood-brain barrier formation, such as neurotransmitter uptake and release, control of ionic homeostasis and physical interaction with blood vessels which enables the regulation of blood flow and metabolism (Chiareli et al. 2021; Farhy-Tselnicker et al. 2021). Multiple studies in astrocytes demonstrated local mRNA translation in distal compartments, including fine processes that contact synapses. We discuss these studies, focusing on how they support the known roles of astrocytes in the modulation of synaptic plasticity.

#### Effects on synaptic plasticity via glial signaling pathways

Biochemical signaling is one of the prime ways through which glial cells can modulate neuronal synaptic plasticity, and mRNAs of various signaling molecules have been identified to be localized to astrocytic protrusions. The mRNAs encoding Ras-related protein (Rab13), Plakophilin-4 (Pkp4), Ankyrin Repeat Domain 25 (Ankrd25), and inositol polyphosphate-1-phosphatase (Inpp1) have all been shown to be localized to the protrusions of primary astrocytes but also in a mouse astrocyte cell line, C8-S (Thomsen and Lade Nielsen 2011).

All these proteins have been shown to be important for various aspects of synaptic plasticity. Rab13 is known to regulate neurite outgrowth (Sakane et al. 2010) and to stabilize myelinating glia-axon contacts (Adriani et al. 2006). Though much less is known about the roles of Rab family proteins in glia, the observations related to Rab13 could suggest that Rabs have bilateral roles, and their expression and localization is important for their correct function in the aforementioned processes both in neurons and glia (Ng and Tang 2008). Pkp4 is a signaling molecule known to modulate cellular adhesion and cytoskeletal remodeling by regulating actin-dependent cellular processes via Rho GTPases, and participating in processes such as neurite outgrowth (Keil et al. 2013). Ankrd25 is a protein which participates in actin stress fibers formation by regulating the Rho signaling pathway (Zhu et al. 2008). Ankrd25 has been shown to be in the top 20 up-regulated genes in frontotemporal lobar degeneration with ubiquitinated inclusions, a type of neurodegenerative disease which causes progressive decline in behavior and executive function (Chen-Plotkin et al. 2008). Polymorphisms in Inpp1, an enzyme involved in the phosphatidylinositol signaling pathway, have been associated with suicidal behavior in bipolar patients (Jiménez et al. 2013), and autism (Serajee et al. 2003).

It is tempting to speculate that the effects related to synaptic plasticity and neuropsychiatric disorders observed in the studies described above are caused by the functions of astrocytes, as opposed or in addition to neurons. Such distinctions are not well studied, but the presence of those RNAs at the astrocyte periphery suggests that this is a promising avenue for future research.

#### Modulation of neuronal function by cytoskeleton remodeling and cell–cell adhesion

One of the well-known ways in which astrocytes modulate synaptic plasticity is via adhesion molecules and cellular junctions. The extracellular domains of adhesion proteins create structural support to bridge the synaptic cleft, and through their intracellular domains, they directly and indirectly link to the actin cytoskeleton, which in turn allows for the dynamic remodeling of the adhesive contacts (Saint-Martin and Goda 2022). Therefore, the dynamic remodeling of the astrocytic cytoskeleton is an interesting consideration in the context of synaptic plasticity.

##### GFAP

GFAP is a type III intermediate filament (IF) protein, an element of the cytoskeleton involved in neuron-glia contacts and the formation of the blood-brain barrier. GFAP modulates astrocyte–neuron crosstalk and can affect the efficacy of the CNS synapses; it is suspected to be required for communications between Bergmann glia and Purkinje cells during long-term depression (LTD), a type of synaptic plasticity at excitatory synapses in the cerebellar cortex thought to be a critical cell level mechanism for motor learning (Hirano 2013).

In one of the earliest studies of mRNA localization in glia, GFAP mRNA was shown to be targeted to the processes of Type-II astrocytes in culture (Medrano and Steward 2001). GFAP mRNA localization requires intact microtubules in the cytoplasmic processes, suggesting a microtubule-mediated mRNA transport mechanism (Medrano and Steward 2001). Furthermore, different GFAP isoforms were found to localize to distinct astrocytic subcellular compartments of mouse primary astrocytes. GFAPα mRNA showed preferential localization to the protrusions compared to GFAPδ mRNA which was localized to the soma, which for GFAPα mRNA was determined by varying 3′-exon sequences present in these isoforms (Thomsen et al. 2013a).

A study of GFAP mutant mice revealed that cerebellar LTD is deficient and eyeblink conditioning are significantly impaired without any detectable deficits in motor coordination tasks (Shibuki et al. 1996).

More sophisticated methods were also used to study GFAP mRNA localization in astrocytes, including cell culture in Boyden chambers, allowing the physical separation and sequencing of astrocyte soma from its cytoplasmic projections (Thomsen and Lade Nielsen 2011). These approaches further emphasized the importance of GFAP in the regulation of synaptic plasticity; it was shown that GFAPα and GFAPδ mRNAs both localize to astrocytic processes, and their distributions are aberrant in a mouse model of Alzheimer's disease, where they do not localize to their usual compartments (Thomsen et al. 2013a).

##### Nestin

Nestin is an intermediate filament protein mostly expressed in neurons, where it participates in modulating the radial growth of the axon (Bott et al. 2019). Both Nestin protein and *nestin* mRNA localizes to the astrocytic protrusions (Thomsen et al. 2013b). Interestingly, *nestin* mRNA localization is dependent on Fragile X mental retardation syndrome proteins Fmrp and Fxr1, and its 3′-UTR is sufficient to mediate the localization.

These results suggest that *nestin* mRNA might be actively transported and locally translated in the astrocytic periphery. A fine regulatory mechanism at the mRNA level exists to control the localization, with potential importance for astrocyte functions during brain development and maintenance. It has been hypothesized that *nestin* mRNA localization and local translation could be responsible for creating the required local environment with optimal conditions for modulation of the intermediate filament assembly in the early onset of astrocytic protrusion formation. Such protrusions are likely to have direct consequences for astrocytic morphology, and their interactions with neurons, leading to modulation of synaptic plasticity.

#### Regulation of membranes, ions and neurotransmitters

Neurotransmitter uptake and release, and the maintenance of ionic homeostasis are some of the most canonical and well-studied functions of astrocytes related to their role in modulating synaptic plasticity. Moreover, a lot of these processes occur at the cellular membranes and involve ionic channels embedded in the membranes. These roles appear to be well supported by a few studies which have analyzed the local population of mRNA in astrocyte processes at genome scale.

One of these studies found that transcripts localized to perisynaptic astrocytic processess (PAPs) had an overrepresentation of genes mediating glutamate and GABA metabolism, consistent with PAP functions of glutamate transport and metabolism like *Slc1a2*, *Slc1a3*, and *Glul* (Sakers et al. 2017). mRNA coding for enzymes in a pathway for biosynthesis of unsaturated fatty acids have also been found, indicating that local regulation of fatty acid generation might be necessary for the expansion of astrocytic membrane in concert with the cytoskeletal remodeling processes, which was also supported by the identification of several transcripts coding for motor and cytoskeletal proteins, like *Kif1c* and *Myo1D.* Transcripts of genes known to regulate synapse number, like *Mertk* and *Thbs4,* were identified too. Translation of one of these, *Sparc*, which is a negative regulator of excitatory synaptogenesis, was also detected locally. Astrocytes could play a significant role in regulating synapse formation and elimination. Moreover, the authors showed that the transcripts which are enriched in PAPs have longer 3′UTRs and are more highly expressed than other transcripts that are absent from PAPs as classified by the authors (“PAP-depleted transcripts”).

Similar results were observed for the blood-brain barrier astrocytes, where the examination of translation in the astrocyte distal endfeet resulted in the identification of an astrocyte “endfeetome,” which consists mainly of proteins destined to cell membranes and for secretion (Boulay et al. 2017). The study purified astrocyte endfeet transcripts; these endfeet are the parts of the astrocytic membranes that directly contact brain blood vessels, and therefore participate in the formation of the blood-brain barrier. Moreover, some of the mRNAs enriched in the endfeet TRAP (translating ribosome affinity purification) libraries, when compared to whole astrocyte TRAP libraries, were found to be mRNAs related to ionic transport (*Slc7a5*, *Slc22a6*), calcium ion binding (*Spock2)*, and cell–cell junctions (*Gjb2*). Another interesting observation made was the demonstration of the presence of smooth and rough endoplasmic reticulum and the Golgi apparatus in astrocyte perivascular processes and endfeet. These observations indicate that local maturation of membrane and secreted proteins is possible in those distal compartments.

#### Regulation of protein translation for synaptic plasticity

Further refinement of the PAP-TRAP method showed that some of the most abundant transcripts in peripheral astrocyte processes (PAP) encoded ribosomal proteins, like Rpl4 and Rplp1, pointing to the presence of translation machinery in these compartments (Mazaré et al. 2020). Poly(A) binding protein PABPC1 and the elongation factors eEF1A1 and eEF2 were among some of the other transcripts found in PAPs. However, it is not clear how these mRNAs contribute to the distal ribosomal pool in the polarized cells. The presence of transcripts encoding ribosomal proteins is a significant consideration in the field of neuronal local translation, and in fact translation distal to the nucleus in any polarized cell. Ribosomal biogenesis is a process known to occur in the nucleolus, and therefore the presence of these ribosomal mRNAs is counterintuitive at the cellular periphery. It could, however, be explained by recent findings which showed that ribosomal proteins locally synthesized in axons join existing axonal ribosomes in a nucleolus-independent fashion known as ribosomal remodeling (Shigeoka et al. 2019). These findings point to the possibility that ribosomal protein translation occurring in distal compartments of polarized cells serves to maintain and modify local ribosomal function (Fusco et al. 2021).

The studies described above suggest an elegant model in which the machinery required for local translation of any relevant mRNAs at the periphery of elongated cytoplasmic projections in any polarized cells, is itself locally translated from mRNAs previously transported to the periphery. Such translation could include de novo assembly of translational complexes or replacement of damaged proteins within the translation complexes, or both, and has been suggested both for astrocytes (Mazaré et al. 2020) and for radial glia (Agrawal and Welshhans 2021).

#### Astrocytes and mRNA localization: a summary

A recent review summarizes the current knowledge of astrocytic transcriptomes and translatomes, listing additional examples of localized transcripts (D'Arcy and Silver 2020). However, there are likely to be many more undiscovered localized transcripts in astrocytic processes as additional cell models are developed and targeted detection approaches are applied more widely.

### Oligodendrocytes and myelin are important for synaptic plasticity

One of the early observations of localized glial mRNAs was in protrusions of Schwann cells and oligodendrocytes (Martone et al. 1998). Although most protein synthesis occurs in the perinuclear regions near the cell bodies, local translation of prelocalized mRNAs takes place at cytoplasmic processes of Schwann cells and oligodendrocytes (Colman et al. 1982; Gould and Mattingly 1990). The myelination of motoneuron axons by Schwann cells is critical for insulation of the electrical signal over large distances. Interestingly, myelin is now also thought to be important for regulating synaptic plasticity (Fields 2005). It is well known that myelination increases when, for example, animals are raised in an enriched environment (Juraska and Kopcik 1988; Sánchez et al. 1998); practicing piano as a child also increases myelination (Bengtsson et al. 2005). Clearly, higher levels of myelination appear to be corelated with synaptic plasticity, yet no studies directly linked the two mechanisms despite the existence of a breadth of literature focused on mRNA localization for correct myelination. It could be hypothesized therefore that the regulation of mRNA localization in Schwann cells can impact not only myelination but also synaptic plasticity.

#### Myelin basic protein mRNA localization is required for correct myelination

In the early stages of the myelination process, an mRNA binding protein, myelin basic protein (MBP), accumulates near the nucleus. However, as myelination proceeds, MBP mRNA also localizes at distal cytoplasmic processes (Trapp et al. 1987; Landry et al. 1994). Similar mRNA distribution has been shown for astrocytes and radial glia in the embryonic brain for the glial fibrillary acidic protein (GFAP) (Sarthy et al. 1989; Landry et al. 1994). A specific 3′-UTR region of the MBP mRNA is required for transport and localization and was later named “A2 response element” (A2RE) as it binds to the heterogenous ribonucleoprotein particle A2 (hnRNP A2). hnRNAP A2 mediates the transport of MBP mRNA along the microtubule cytoskeleton toward the myelin-producing compartments in the oligodendrocyte processes (Ainger et al. 1993, 1997; Munro et al. 1999a). Subsequently, the mRNA for another myelin-localized protein, myelin-associated oligodendrocytic basic protein (MOBP), was also found to contain a similar localization signal to A2RE (Gould et al. 2002).

#### Correct myelination depends on mRNA localization by RNA binding proteins

**FIGURE 2.**
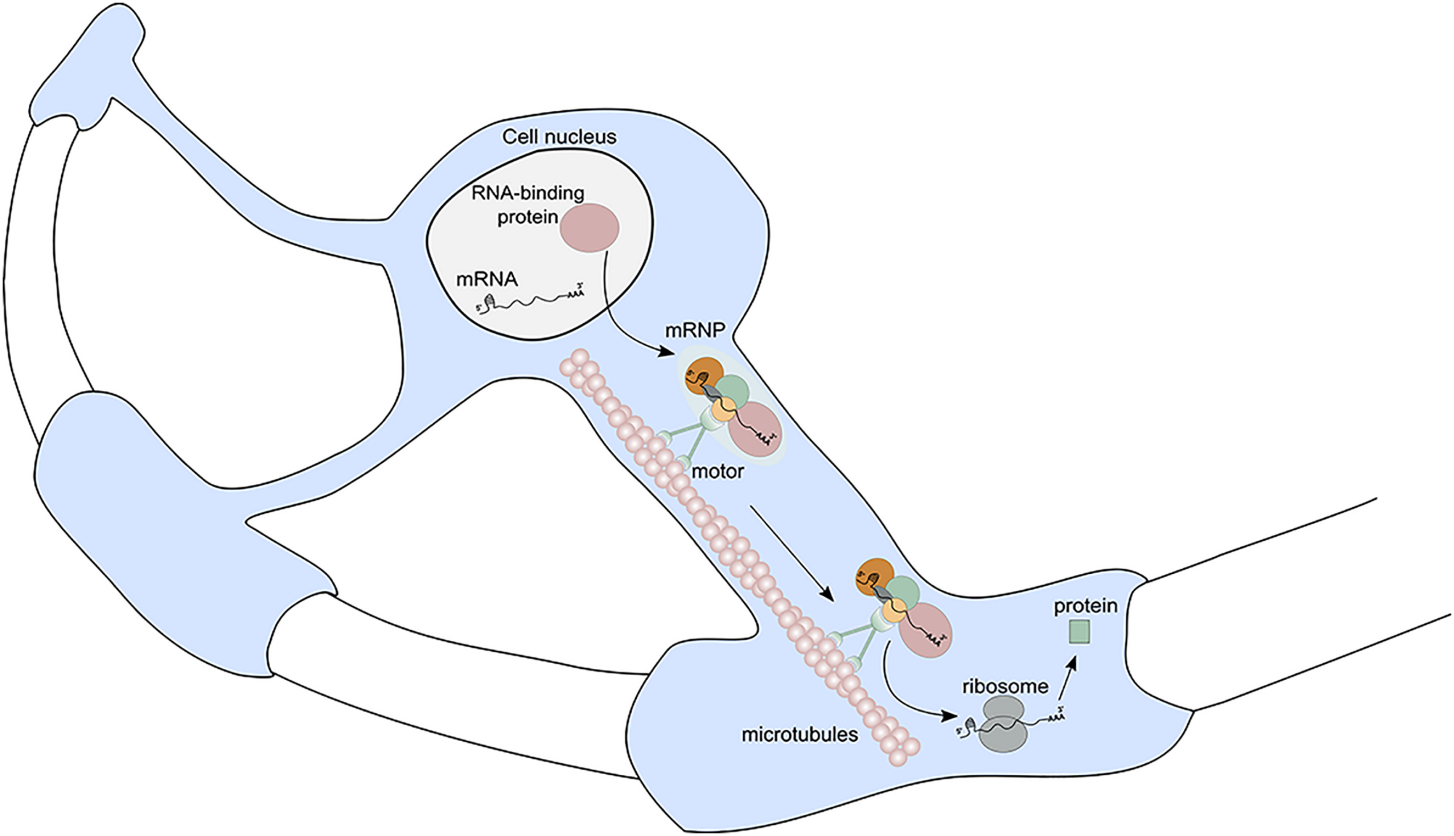
A generalized model of mRNA trafficking in glia. A nascent mRNA is bound in the nucleus by an mRNA-binding protein, like hnRNP A2 in oligodendrocytes, through a 3′UTR response element. Other well-known RBPs include zipcode-binding protein ZBP1 or IMP-1 in neuronal growth cones and hippocampal dendrites; whether these signals participate in trafficking of other mRNAs than that of MBP in glia is not known, yet plausible. The mRNA–RBP complex is next shuttled out of the nucleus and assembled into messenger ribonucleoprotein (mRNP) granules in the cytoplasm. Those are transported by a motor protein, like kinesin, along microtubules toward the site of required translation, where protein can be made using localized translation machinery.

Further studies of the mechanism by which mRNAs are transported toward the myelinating glial cell periphery in oligodendrocytes showed that the movement of MBP mRNA in oligodendrocytes is on microtubules, not microfilaments (Carson et al. 1997). These observations led to the development of a generalized model of mRNA localization in glia (Fig. 2) in which MBP mRNAs interact with hnRNP A2 by its A2RE, which causes the shuttling of hnRNP A2 out of the nucleus. In the cytoplasm, the hnRNP A2 with its bound mRNA is assembled into granules composed of messenger ribonucleoproteins (mRNPs) that are transported to the myelin compartment by a dual kinesin-dynein motor mechanism. It has been shown that translation machinery is present in the processes of oligodendrocytes (Barbarese et al. 1999), and hypothesized that the trafficked mRNA is translationally repressed during the transport, and activated once it reaches its destination.

The question of detailed regulation of mRNA localization in oligodendrocyte myelination continues to be explored as it represents an important point of regulation of myelin translation and secretion (for reviews, see Martin and Ephrussi 2009; Percipalle 2014; Neriec and Percipalle 2018). The disruption of hnRNP A2 binding to MBP mRNA by point mutations in A2RE causes a significant disruption in mRNA trafficking (Munro et al. 1999b). Motor proteins like the kinesin Kif1b are necessary for the MBP mRNA localization to processes of myelinating oligodendrocytes in zebrafish (Lyons et al. 2009). The outgrowth of some of the longest axons in both the PNS and CNS requires Kif1b, and disruption of Kif1b motor function leads to ectopic myelin in oligodendrocyte cell bodies (Lyons et al. 2009).

#### QKI protein could be a master regulator of mRNA localization in oligodendrocytes and other glia

MBP mRNA is also bound by Quaking RNA binding protein (QKI). The *QKI* gene exists as three separate protein isoforms termed QKI-5, with nuclear localization and QKI-6, and QKI-7, which localize to the cytoplasm (Kondo et al. 1999). In fact, these isoforms display a network of auto- and cross-regulation of QKI protein isoforms by controlling alternative splicing of the QKI mRNA, but also other mRNAs (Fagg et al. 2017).

It has previously been shown that QKI is required for oligodendrocyte development during the process of myelination by regulating MBP mRNA transport (Li et al. 2000; Larocque et al. 2002). Specifically, QKI mRNA binding proteins were shown to bind the MBP mRNAs directly via a 3′-UTR and regulate the MBP mRNA nuclear export. The study examined what happens to MBP mRNA in *quaking viable* (*qk*^*v*^) mice, which are a widely used model for dysmyelination because they exhibit hypomyelination of both the CNS and PNS resulting from spontaneous mutations in the promoter and enhancer regions of the *qk* gene. Nuclear and perikaryal retention of MBP mRNAs was observed in *qk*^*v*^ mice, suggesting that QKI takes part in myelination by regulating the MBP mRNA export and cellular targeting to the periphery (Larocque et al. 2002). These results clearly indicate that MBP mRNA localization is indispensable for proper myelination and neuronal function. Furthermore, it seems likely that a wide variety of localized mRNAs that possess A2RE-like sequences exist and play similarly important roles as MBP mRNA.

However, MBP is far from being the only mRNA controlled by QKI. A recent study identified 120 targets of QKI in the mouse brain (Sakers et al. 2021), 24 of which overlapped with PAP-TRAP data, gathered by the same group (Sakers et al. 2017). Those mRNAs included, again, solute carrier family proteins involved in glutamate (*Slc1a2*, *Slc1a3*, Glul) and GABA (*Slc6a11)* transport and metabolism, membrane dynamics and cell–cell adhesion regulation (*Agpat3*, *Cpe*, *Sptbn1*, *Pcdh1*), *a* cytoskeletal dynamics regulator (*Fam107a*), signaling pathways (*Sash1 Ntsr2 Hipk2, Ptprz1*), and neurite outgrowth (*Ndrg2*, *Sparc*). Therefore, QKI appears to be highly expressed and highly important both in oligodendrocytes and in other types of glia. Moreover, some of its localized targets participate in the key processes known to affect glia-neuron crosstalk and potential modulation of neuronal synaptic plasticity by those glia. Hence, QKI could be a key player in facilitating or enabling the local control which glia exert on the neighboring neurons.

#### Other transcripts implicated in synaptic plasticity are present in oligodendrocyte protrusions

MBP is not the only significant mRNA found in the protrusions of oligodendrocytes, but only one study has focused on the whole transcriptome of oligodendrocyte protrusions (Azevedo et al. 2018). Much like in astrocytes, the authors found that ∼30% of transcripts that were enriched in the protrusions of rat brain oligodendrocyte progenitors coded for proteins required for cytoskeleton dynamics (Azevedo et al. 2018), including JMY, the Junction-mediating and -regulatory protein. The study showed that upon JMY knockdown, the progenitors fail to develop the typical branched morphology and cannot make extensive contacts with neurons, both of which are required for myelination. Another study focusing on the transcriptome of mouse myelin showed that mouse CNS myelin, which is produced by oligodendrocytes, depends on the specific targeting of selected mRNAs to the myelin compartments (Thakurela et al. 2016). Moreover, several transcripts coding for enzymes related to lipid metabolism were found to be enriched in these myelin regions, but some of the most abundant transcripts identified had no previously known relation to CNS myelination, such as *Plekhb1*, *Bcas1*, *Trp53inp2*, and *Ptgds* (Thakurela et al. 2016).

### Microglial motility and phagocytic functions contribute to synaptic plasticity

Microglia have been known to be involved in the modulation of synaptic plasticity for some time. Three roles for microglia in plasticity have been suggested: modification of the perisynaptic environment by ECM (extracellular matrix) proteolysis, dendritic spine remodeling and engulfment of dendritic spines and axon terminals (Tremblay and Majewska 2011). Many of the microglial roles in synapse formation and elimination both in health and in disease could be compared to the roles of tripartite synapse glia; reviewed elsewhere (Tremblay 2012; Morris et al. 2013; Wu et al. 2015; Zaki and Cai 2020; Andoh and Koyama 2021).

Interestingly, microglia were found to engulf pre-synaptic terminals in response to neural activity (Schafer et al. 2012), and it has been suggested that the synapses that are destined to be engulfed express C3b-opsonization, which is recognized by microglia, the only known CNS cells to express the C3 receptor (Gasque et al. 1998; Stevens et al. 2007). Therefore, microglia have a key role during triaging for synaptic pruning or maintenance of synapses, which again emphasizes that they might need rapid production of cytoskeletal proteins at their periphery. It will be interesting to follow future discoveries of the signals and other molecular mechanisms involved in these important functions of microglia.

Despite the rising general interest in microglia, particularly in the context of a deadly class of brain tumors called glioblastoma (Geribaldi-Doldan et al. 2020; Liu et al. 2021) mRNA localization has barely been studied in these cells. One very recent exception is a preprint of a study that identified 258 mRNAs localized to microglial protrusions, peripheral microglia processes (PeMPs) (Vasek et al. 2021). Those transcripts coded for proteins involved in phagocytosis and immune processes as well as motility, suggesting active roles in pathogen defense and injury related processes, which strongly supports the importance of those localized mRNAs in the aforementioned roles of microglia in synaptic plasticity. Rpl4 mRNA coding for a ribosomal protein has also been found in microglial processes (Oudart et al. 2020). These unexpected observations raise the possibility that proteins required for local translation might themselves have localized mRNAs which are also translated locally or participate in the remodeling and repair of the existing pool of ribosomes, as explained previously (Shigeoka et al. 2019). Undoubtedly, more studies of local translation in microglia are bound to emerge in the future, focusing not only on their involvement in synaptic plasticity, but also on their immune roles in neurodegeneration, injury, and cancer.

### Radial glia control early developmental plasticity

Radial glia are stem cells of the developing nervous system with unique elongated morphology supporting their roles in guiding the radial migration of new-born neurons. They are also known to differentiate into various types of CNS cells, such as neurons, astrocytes, and oligodendrocytes (Campbell and Götz 2002). Radial glia are known to exhibit polarized mRNA transport within their specialized radial morphology adapted to support neuronal migration (Pilaz et al. 2016). It is also possible that radial glia locally regulate various facets of neuronal development, including synapse formation (Allen and Lyons 2018). N-Cadherin accumulates at the site of interaction of radial glia with cortical neurons, and axons project at the opposite side of the neuron from the contact site (Xu et al. 2015). It seems reasonable to hypothesize that these polarization events involve mRNA transport and localized translation in both glia and neurons.

Radial glia have been a powerful system for investigating mRNA localization, and transcripts localized to their endfeet were also examined (Pilaz and Silver 2015; Pilaz et al. 2016, 2020; Roko Rasin and Silver 2016; D'Arcy and Silver 2020). Among these localized transcripts are mRNAs known to be bound by an RNA binding protein, Fragile X mental retardation protein (FMRP), including microtubule-associated proteins and several signaling molecules. It has been shown that mRNA and mRNA-binding proteins, like FMRP, are trafficked in basal processes of radial glia, likely via microtubule-based transport. Moreover, many of the transcripts bound to FMRP were ones with known roles in autism and neurogenesis, coding for signaling and cytoskeletal regulators (Pilaz et al. 2016).

Local gene regulation in radial glia has been the subject of a recent extensive review, and their similarity to phylogenetically connected cells like neurons and astrocytes has been emphasized (D'Arcy and Silver 2020). Moreover, a significant overlap has been shown in the types of localized mRNAs in these cell types, further suggesting that local translation might be important for any type of elongated cell of the nervous system to achieve fine control of signaling and connectivity.

### Perisynaptic Schwann cells express synaptically relevant mRNAs

Perisynaptic Schwann cells (PSCs) are the synapse-associated glia of the PNS and therefore intimately associated with the synaptic activity of neuromuscular junction synapses. The examination of bulk transcriptomes from FACS-sorted PSCs pointed to a high number of transcripts related to glutamate receptor function, axon guidance, and synaptogenesis (Castro et al. 2020). By cross-referencing their data, the authors identified enriched genes with functional roles in PSCs, some of which include transcripts related to synaptic pruning, synaptic activity modulation, and myelination. These findings again suggest that glial mRNA localization can be an active player in synaptic plasticity.

## BIOLOGICAL SIGNIFICANCE AND THE APPLICATION OF ADVANCED TECHNOLOGIES

Despite the considerable volume of evidence that mRNA is localized in glial projections in many contexts, with a few notable exceptions, in the vast majority of cases localization has not been shown to be functionally essential. One notable study presented an observation that mRNA distribution and local translation in perisynaptic astrocytic processes (PAPs) in mice changes after fear conditioning (Mazaré et al. 2020). Although the study highlights the molecules that are present in glial projections, the data does not address whether changes in mRNA distribution are functionally significant. Translation of the glial protein *Sparc*, a negative regulator of excitatory synaptogenesis, was also detected locally in PAPs, but no studies were done to prove that excitatory synaptogenesis is impaired or impossible without *Sparc* mRNA in astrocytic projections (Sakers et al. 2017). Despite the lack of many other examples, there is a compelling intellectual argument that mRNA localization and targeted translation are needed at the tips of glial projections to influence adjacent synapses rapidly.

Given that local responses of astrocyte and PSCs are likely to be rapid, it has been hypothesized that mRNA localization and local translation at the astrocyte and PSCs protrusions likely play a role akin to what occurs in neurons. Neuronal studies involving mRNA localization and targeted translation have not only been more numerous than the equivalent glial work but have also involved a more diverse repertoire of advanced technologies. Those studies have made use of modern biochemical and genome-wide approaches, as well as single molecule and super-resolution microscopy methods.

Neuronal mRNAs have previously been successfully tagged with the MS2 loops which results in mRNA decorated with fluorescent proteins fused to the MS2 coat protein (MCP) (Bertrand et al. 1998; Weil et al. 2010; Park et al. 2014). MS2–MCP enables live tracking of mRNAs transport in neurons, as well as observing the activity-dependent behavior of transcripts of interest (Hoppe and Ashe 2021), and has been successfully used in radial glia (Pilaz et al. 2016). However, as mentioned above, radial glia are a stem cell type and some radial glia do differentiate into neurons. It would be interesting to deploy the MS2–MCP in mature glia such as astrocytes or oligodendrocytes to follow changes in mRNA localization in living cells, upon electrical or chemical stimulation.

Another prominent technique is single molecule fluorescence in situ hybridization, or smFISH, and its related techniques. smFISH remains a gold standard for visualizing mRNA localization in fixed samples with high sensitivity and resolution. This technique has also begun to be applied to glial cells in intact tissues (Titlow et al. 2018, 2022). smFISH could be utilized to further describe the mRNA repertoire of the glial projections, particularly in distinct spatial compartments using super-resolution microscopy. smFISH is a very efficient technique to confirm the observations made by sequencing studies, and even quantify the numbers of mRNAs of interest present in the periphery. Hybridization chain reaction (HCR) and RNAScope are complementary FISH methods to smFISH. They offer signal amplification, which allows for easier imaging of stronger signal, although usually at the expense of single molecule quantitation (Wang et al. 2012; Tsuneoka and Funato 2020). Multiple smFISH or HCR probes can be used simultaneously to multiplex the detection of many distinct transcript types. This enables the visualization of mRNA localization in the context of the ultrastructural morphology of synaptic structures by electron microscopy.

Importantly, observing localized mRNA is not equivalent to demonstrating its functional significance. To explicitly address the functional significance of the localization of specific mRNAs to the glial periphery for influencing synaptic plasticity, it would be necessary to visualize mRNA translation in living glia, while assaying synaptic plasticity. mRNA translation has been visualized in living neurons using MS2–MCP combined with an orthogonal labeling system (Halstead et al. 2015; Wu et al. 2016). Imaging translation in living glia would be particularly powerful if used pre and post-stimulus, such as during fear conditioning, in order to image mRNA transport and translation changes caused by neuronal stimulation. Other existing tools for visualizing translation in live cells, include the SunTag system which has been used both in neurons and in glia (Wu et al. 2016). However, the paper does not include any functional observations related to the translation of mRNA in glia in response to neuronal activity. Ideal experiments would involve using the SunTag system to monitor translation live pre and post- stimulus, like suggested above for the MS2–MCP system. However, carrying out such experiments in living animals is still technically very difficult, and therefore initial progress could be made in cell culture, with the usage of neuronal and glial coculture and potential deployment of simultaneous or parallel calcium and voltage imaging.

Finally, there are many tools that automate the quantification of glial mRNA molecules in microscopy images. These include, tools like AstroDot and AstroStat (Oudart et al. 2020) as well as FISH-quant (Mueller et al. 2013; Imbert et al. 2022) for automated detection and quantification of mRNA foci in smFISH experiments. All these diverse and powerful tools can certainly be, in principle, also applied to glia, like neurons. Only then will the experimental analysis of the mechanism and function of glial mRNA transport and localized translation progress rapidly begin to catch up with the equivalent studies in neurons.

## OPEN HORIZONS

In this review, we describe several examples of mRNA localization and localized translation in a diverse range of glial subtypes, which represent “the tip of the iceberg.” The following is a nonexhaustive list of important and fascinating questions that will no doubt be tackled by the field in the future.

### Are the molecular mechanisms that drive mRNA localization in other types of glia similar to, or different from those observed in oligodendrocytes?

Recent research supports the idea that RNA interactions with RBPs, which have the capacity to regulate RNA localization in one cell, can predictably regulate localization in other cell types with vastly different morphologies (Goering et al. 2022). It is quite probable that these mechanisms would therefore be conserved and any cell with long processes could localize similar mRNAs, for example, mRNAs coding for cytoskeletal elements or ribosomes, to the cell periphery. Using genetic techniques such as selective ARE mutations, or smFISH and immunofluorescence, the same key players could be identified and their localization and role examined in astrocytes or microglia, for example. Nevertheless, it is also possible and indeed expected that at least some localized transcripts at the distal periphery of different cell types will be distinct, or that different transcripts are selectively translated at the periphery of different cells.

### Are the mechanisms of mRNA transport and localized translation conserved between different species?

Basic neuronal mechanisms and functions appear to be well conserved in neurons from *Drosophila* to humans, so it is probable that localized translation could also be widely conserved. Certainly, the RBPs implicated in localization, such as Imp/ZBP and microtubule associated molecular motors are highly conserved, although it is not clear whether the mRNA localization signals are equally conserved.

### How close and universal is the interplay between glia and synapses?

What are the molecular mechanisms responsible for activity-regulated gene expression in PSCs? It would be intriguing to know how terminal glia near neuromuscular junctions (NMJ) can differentially localize mRNAs to glial cytoplasmic projections near the NMJ synapses versus other parts of the cell. However, to our knowledge, no publications exist exploring this topic. All these questions are fascinating but will require considerable future work by many labs to answer, especially considering how difficult these glia are to separate from the muscle and from the axon terminal; techniques like FACS slowly start to provide viable solutions to such problems (Castro et al. 2020).

### Is mRNA localization dysregulated in neurodegenerative and neuropsychiatric disorders?

The prior work in neuronal mRNA localization and RBPs in neurodegenerative and neuropsychiatric disorders raises the question of whether mRNA localization in glia has a similar importance for such diseases (Blanco-Urrejola et al. 2021). Exploring such questions will require the ability to recapitulate mutations that cause human disease in models for the disease in which the mutations can be introduced very specifically in distinct glial subtypes. Time will tell whether many neurodegenerative and neuropsychiatric diseases are caused by glial specific mRNA localization and localized translation.
